# Lipoyl Synthase in Focus: Radical SAM Chemistry and the Biosynthesis of Lipoic Acid

**DOI:** 10.1021/acsbiomedchemau.6c00104

**Published:** 2026-07-25

**Authors:** Olga E. Esakova, Caitlin D. Palmer, Douglas M. Warui, Erin L. McCarthy, Squire J. Booker

**Affiliations:** † Department of Chemistry, 6572 The Pennsylvania State University, University Park, Pennsylvania 16802, United States; ‡ Department of Chemistry, School of Arts and Sciences, University of Pennsylvania, Philadelphia, Pennsylvania 19104, United States; § Department of Biochemistry & Molecular Biology, The Pennsylvania State University, University Park, Pennsylvania 16802, United States; ∥ Department of Biochemistry and Biophysics, Perelman School of Medicine at the University of Pennsylvania, Philadelphia, Pennsylvania 19104, United States; ⊥ Howard Hughes Medical Institute, Chevy Chase, Maryland 20815, United States

**Keywords:** S-adenosylmethionine, radical, lipoic acid, iron−sulfur cluster, protein maturation, NfuA, lipoyl synthase

## Abstract

Lipoic acid (1,2-dithiolane-3-pentanoic acid or 6,8-thioctic acid) is a sulfur-containing cofactor essential for central metabolism, where it functions in oxidative decarboxylation reactions catalyzed by the 2-oxoacid dehydrogenase complexes and glycine degradation catalyzed by the glycine cleavage system. Beyond its canonical metabolic roles, the redox-active disulfide/dithiol pair of lipoic acid has attracted considerable interest because of its proposed therapeutic relevance in ailments associated with oxidative stress and inflammation, such as Alzheimer’s and Parkinson’s disease. Despite the broad biological importance of this cofactor, the molecular details of its biosynthesis remained elusive for many years because of the unusual chemistry required for sulfur insertion into unactivated carbon centers. Recent advances have subsequently expanded the understanding of this pathway, particularly through mechanistic and structural studies of lipoyl synthase, a radical *S*-adenosylmethionine enzyme that catalyzes sulfur incorporation during cofactor assembly. This perspective highlights current insights into lipoic acid biosynthesis and lipoyl synthase, with an emphasis on emerging mechanistic paradigms, lipoyl synthase auxiliary cluster function and regeneration, and the broader biochemical context of radical-mediated sulfur insertion.

## Graphical abstract

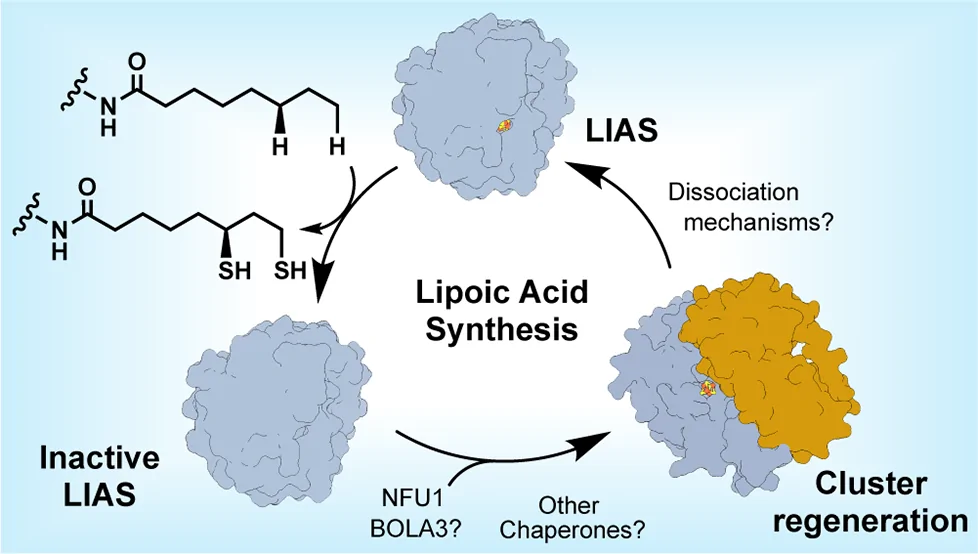

## Introduction

Lipoic acid (1,2-dithiolane-3-pentanoic acid or 6,8-thioctic acid) is an eight-carbon fatty acid that contains sulfur atoms at C6 and C8, which forms a reversible intramolecular disulfide to yield a five-membered dithiolane ring (Figure 1A and B).^1,2^ C6 is the sole stereocenter, with the *R*-enantiomer being formed *in vivo*.^3,4^ In pharmacological contexts, the term α-lipoic acid is used to distinguish it from β-lipoic acid, which bears a C6 sulfenic acid modification and exhibits diminished activity (Figure 1C).^5,6^ Lipoic acid undergoes a reversible two-electron reduction to dihydrolipoic acid (E°’ = −325 mV at 25 °C and pH 7.0)^7^ (Figure 1D), comparable to the NAD­(P)^+^/NAD­(P)H redox couple (E°’ = −318 mV).^8^ Its amphipathic character and redox potential underlie its widely studied antioxidant properties and therapeutic applications in oxidative stress-associated pathologies, such as diabetic neuropathy, Alzheimer’s disease, and Parkinson’s disease.^9–13^

**Figure 1:**
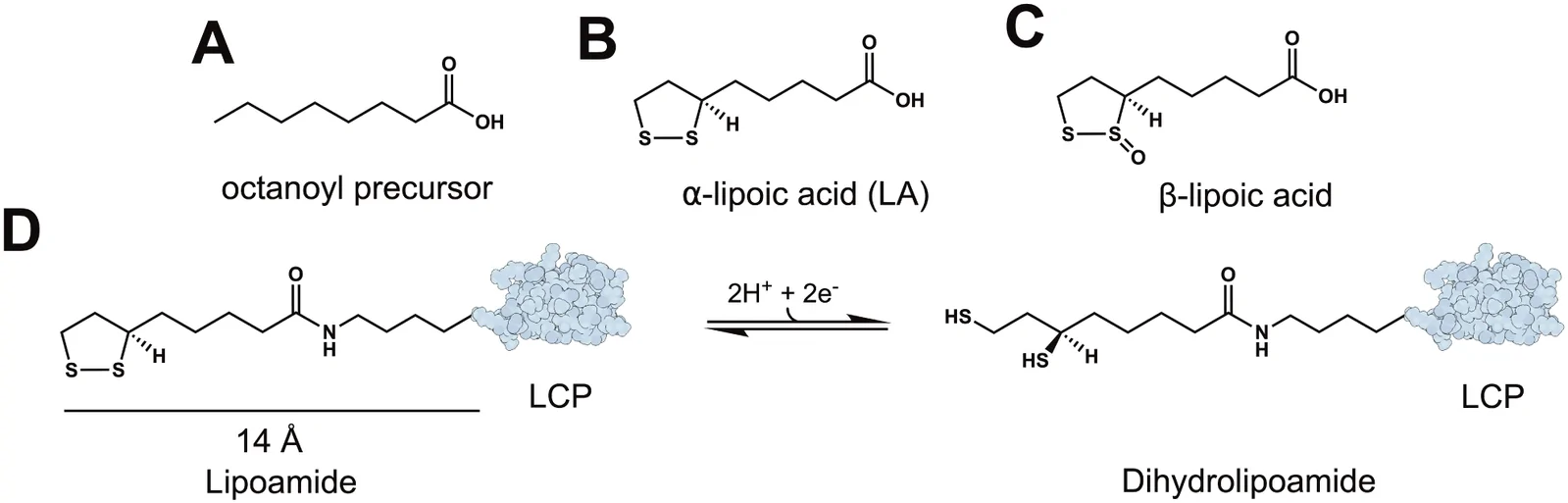
Structure of (A) the octanoyl precursor, (B) α-lipoic acid, (C) β-lipoic acid, and (D) lipoamide in its oxidized (*left*) and reduced (*right*) forms. Reduced lipoamide is also known as dihydrolipoamide. In its biologically active form, LA is covalently attached through an amide linkage to a strictly conserved lysyl residue on an LCP (blue cartoon), producing a flexible 14 Å tether.

Lipoic acid (LA) is rarely found in its free form in the cell, existing predominantly as a covalently bound cofactor (lipoyl cofactor, LC).^14,15^ LA is covalently attached via an amide linkage to a strictly conserved lysyl residue of a lipoyl carrier protein (LCP), forming a 14 Å appendage (Figure 1D) that is often described as a “swinging arm,” which transfers reaction intermediates between spatially distinct active sites. The LC is essential for a class of multienzyme complexes that catalyze oxidative decarboxylation and acyl-group transfer reactions central to primary metabolism. These include the 2-oxoacid dehydrogenase complexes, such as the pyruvate dehydrogenase complex (PDC), the α-ketoglutarate dehydrogenase complex (KDC), the branched-chain oxoacid dehydrogenase complex (BCOADC), the α-ketoadipate dehydrogenase complex (KADC), as well as the glycine cleavage system (GCS).^2,16,17^ In certain bacteria, it is also used to degrade acetoin through the acetoin dehydrogenase complex (ADC).^18,19^ Through these systems, the LC enables substrate channeling and coordinated catalysis at the interface of central carbon metabolism and amino acid catabolism.

With the exception of the ADC, these enzyme complexes are conserved across all domains of life. In eukaryotes, LA-dependent enzymes and the machinery responsible for LC biosynthesis are primarily localized in mitochondria, with additional localization in plastids in plants and in specialized organelles in certain protists, such as *Chlamydomonas reinhardtii*.^16,20,21^ Consistent with its central metabolic role, LA is essential in mammals and most eukaryotes, and defects in LC biosynthesis or its utilization result in severe metabolic disorders.^22^ Despite its structural simplicity, the biosynthesis of LA is chemically demanding, requiring the installation of sulfur atoms into unactivated carbon centers.^23–26^ This transformation is catalyzed by lipoyl synthase (LipA in bacteria; LIAS in humans), a member of the radical *S*-adenosylmethionine (SAM) enzyme superfamily that employs two [4Fe-4S] clusters.^27–29^ Lipoyl synthase initiates the stepwise abstraction of hydrogen atoms from octanoyl substrates and the concomitant formation of C–S bonds, using an auxiliary [4Fe-4S] cluster as the immediate sulfur source.^27,30^ This process entails the sacrificial degradation of the auxiliary cluster during turnover, necessitating mechanisms for cluster regeneration to sustain the catalytic activity. As such, lipoyl synthase operates at the intersection of radical chemistry, sulfur trafficking, and Fe/S-cluster homeostasis.

The requirement for repeated cluster reconstitution distinguishes lipoyl synthase from other radical SAM enzymes and raises fundamental mechanistic questions regarding how Fe/S clusters are assembled, delivered, and recycled under catalytic conditions. Elucidating these processes is essential not only for understanding LA biosynthesis but also for defining broader principles governing metallocluster dynamics in biology. Herein, we examine the current state of knowledge surrounding LC biosynthesis with a particular emphasis on the mechanistic basis of sulfur insertion via lipoyl synthase and the pathways that support Fe/S cluster maintenance during catalysis.

### Pathways for LC Biosynthesis

The biosynthesis and utilization of the LC were first discovered in *Escherichia coli*, where two mechanistically distinct pathways were identified: an exogenous salvage pathway and an endogenous *de novo* biosynthetic pathway (Figure 2, *top*).^16,31^ In the salvage route, free lipoic acid is activated by ATP-dependent adenylation and subsequently transferred to a conserved lysyl residue of lipoyl carrier proteins (LCPs) by lipoate protein ligase A (LplA), yielding the protein-bound cofactor with the release of AMP.^15,32^ This two-step mechanism enables the direct incorporation of environmental lipoate into central metabolic complexes. In contrast, the endogenous pathway constructs the cofactor *de novo* from octanoyl precursors.^33,34^ An octanoyltransferase (LipB) catalyzes the transfer of an octanoyl group from octanoyl-acyl carrier protein (ACP) to the conserved lysine residue of LCPs.^35^ The resulting octanoylated proteins are subsequently modified by lipoyl synthase (LipA), which installs two sulfur atoms at C6 and C8 to generate a functional dithiolane ring.^35,36^

**Figure 2:**
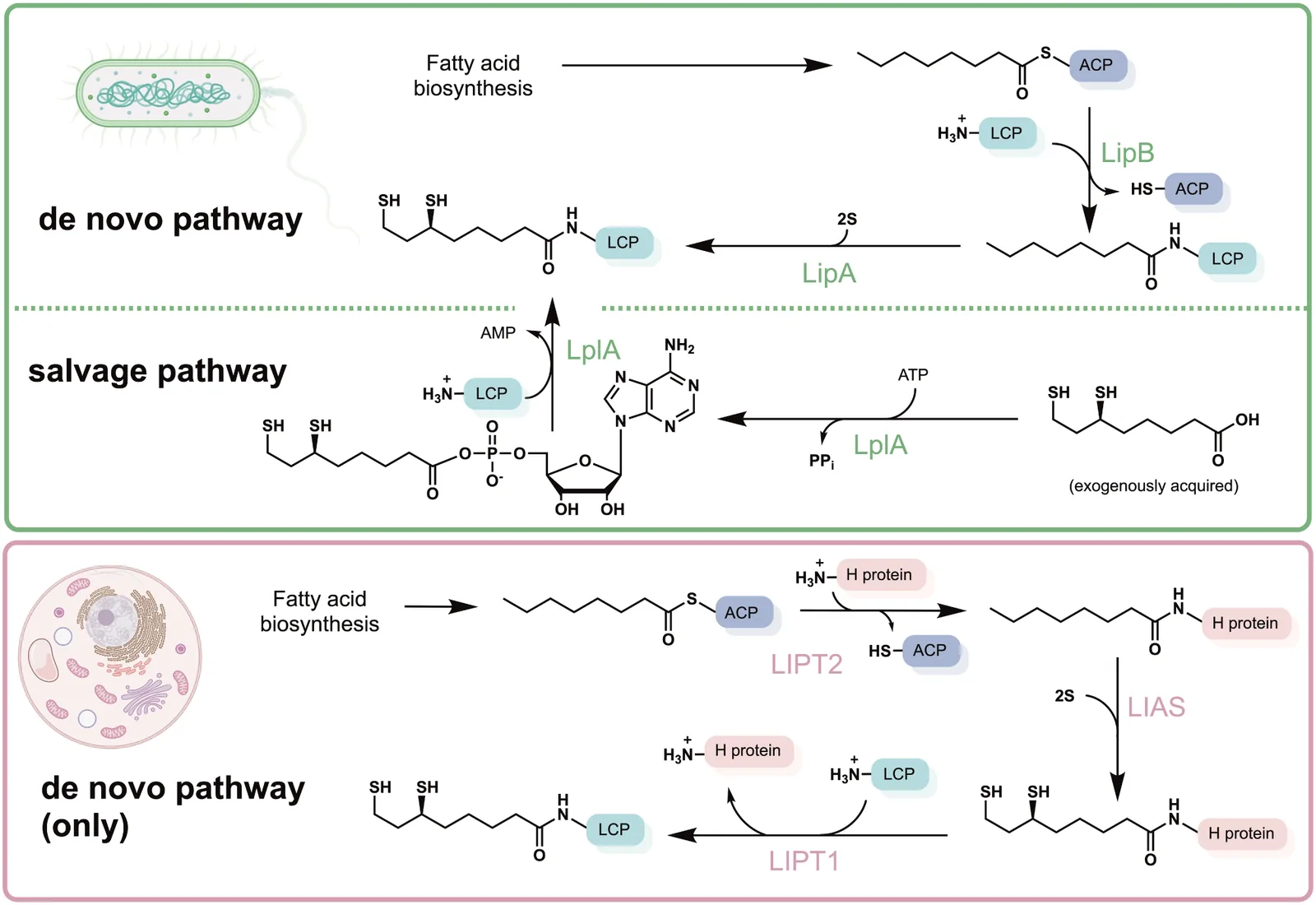
Schematic representation of LC biosynthesis pathways (*top,* green). The *de novo* and salvage LC biosynthetic pathways in *E. coli*. In the *de novo* pathway, octanoyl-ACP, in an offshoot from Type II fatty acid synthesis, serves as a substrate for LipB, which transfers the octanoyl chain to an LCP. LipA then catalyzes the attachment of sulfurs at C^6^ and C^8^ to generate the LC. In the salvage pathway, LA is scavenged from the environment and activated by LplA in an ATP-dependent manner, followed by the addition of an LCP to form the LC. Both processes occur in the cytoplasm. *(bottom*, pink) The *de novo* LC biosynthetic pathway in humans, which is initiated by the transfer of the octanoyl group from ACP1 to the H-protein by LIPT2. Once bound to the H-protein, LIAS attaches two sulfurs to form the LC. Subsequently, LIPT1 distributes the LC from the H-protein to other LCPs. This process is carried out in the mitochondria of humans and other eukaryotic species.

While *E. coli* utilizes discrete salvage and *de novo* pathways, other bacteria, archaea, and eukaryotes exhibit expanded or reorganized lipoylation networks (Table 1). For example, in *Bacillus subtilis*, LC assembly requires four proteins compared to the two in *E. coli*.^37,38^ Lipoyl groups are first synthesized on the glycine cleavage system H-protein before being transferred to other LCPs, reflecting a hierarchical distribution mechanism distinct from that found in *E. coli*. Lipoic acid biosynthesis in the thermophilic archaeon *Thermococcus kodakarensis* (*T. kodakarensis*) proceeds through distinct *de novo* and salvage pathways that differ substantially from those found in bacteria. The *de novo* pathway relies on the radical SAM proteins LipS1 (TK2109) and LipS2 (TK2248), in contrast to bacterial systems that employ only LipA.^39^ A subsequent study demonstrated that a heterodimeric lipoate-protein ligase composed of TK1908 and TK1234 is required for both *de novo* lipoylation and lipoate salvage.^40,41^ During *de novo* biosynthesis, this ligase was shown to attach octanoate to the target protein, which is subsequently converted to lipoyl-protein by LipS1/LipS2.^39,42^ In the salvage pathway, the TK1908/TK1234 ligase complex directly attaches exogenous lipoate, bypassing the sulfur insertion step.^41^

**Table 1:** LA Biosynthesis Pathways in Model Species

**Organism**	***E. coli***	***B. subtilis***	***S. cerevisiae***	***T. kodakarenisis***	***H. sapiens***
Presence of salvage pathway?	Yes	Yes	No	Yes	No
Presence of *de novo* pathway?	Yes	Yes	Yes	Yes	Yes
Initial substrates	Free LA; octanoyl-ACP; 6-thiooctanoic acid; 8-thiooctanoic acid	Free LA; octanoyl-ACP	octanoyl-ACP	Free LA; octanoic acid	octanoyl-ACP1
Lipoate ligase	LplA	LplJ	N.A.^a^	TK1908/TK1234 protein complex	N.A.^a^
Lipoyl transferase	LplA	LipL	Lip3	TK1908/TK1234 protein complex	LIPT1
Glycine H-protein	GcvH	GcvH	Gcv3	GcvH	GSCH
Octanoyl transferase	LipB	LipM; LipL; Lip2	Lip2	--^b^	LIPT2
Lipoyl synthase	LipA	LipA	Lip5	LipS2; LipS1	LIAS
Lipoylation pattern	C6 followed by C8	C6 followed by C8	C6 followed by C8	C8 followed by C6	C6 followed by C8

a N.A. = not applicable; does not contain an LC salvage pathway.

b Acyl carrier protein homologues have not been found in archaeal genomes, and the TK1908/TK1234 protein complex does not utilize octanoyl-CoA as a substrate. Therefore, it is unclear whether an octanoyl transferase exists in these systems.

In *Saccharomyces cerevisiae*, only the *de novo* pathway is present. The octanoyltransferase Lip2 modifies the H-protein (Gcv3), which is thiolated by Lip5 (LipA homolog) to generate lipoylated Gcv3.^43^ Lipoyl transfer to additional mitochondrial complexes, including PDH and AKGD, is mediated by the lipoyl transferase Lip3 in a mechanism similar to that observed in *Staphylococcus aureus*.^44^ Unlike bacterial LplA, Lip3 lacks the ATP-dependent activation of free lipoate and instead operates as a redistribution enzyme that acts on protein-bound intermediates. Consistent with this organization, exogenous lipoic acid does not restore lipoylation defects in *lip2Δ, lip3Δ,* or *lip5Δ* strains, and delayed growth was observed when a *lip2Δ* strain was grown on medium containing glycine as the sole nitrogen source,^43^ suggesting a GCS lipoylation deficiency. Heterologous expression of bacterial LplA is required to establish salvage capability,^43^ indicating the absence of an intrinsic salvage pathway in yeast.

In mammals, lipoylation is likewise restricted to endogenous pathways, and earlier proposals of dietary lipoate utilization have been revised.^45^ Although exogenous lipoic acid enters the circulatory system and tissues via dietary supplementation, isotopic tracing demonstrates rapid metabolic degradation via β-oxidation without detectable incorporation into protein-bound LC.^46,47^ Thus, mammals lack a functional salvage pathway for LC assembly. Instead, mitochondrial lipoylation is mediated by a conserved three-enzyme system comprising the octanoyltransferase (LIPT2), lipoyl synthase (LIAS), and lipoyltransferase (LIPT1) (Figure 2, *bottom*). Genetic disruption of any of these components in mice,^48^ humans,^24,49–51^ or *Caenorhabditis elegans* ^52,53^ results in severe metabolic dysfunction that is not rescued by lipoic acid supplementation, underscoring the exclusivity of the endogenous pathway.

In humans, LC biosynthesis occurs in mitochondria and is initiated by the LIPT2-mediated^51,54^ transfer of an octanoyl group from acyl carrier protein (ACP1) to GCSH (H protein).^55^ The resulting octanoylated intermediate is the exclusive substrate for LIAS, which installs two sulfur atoms to form the mature LC.^56–59^ Recent structural studies of human LIAS and LIAS-GCSH complexes revealed a strong conservation-of-fold architecture across bacterial (e.g., *M. tuberculosis* and *T. elongatus*) and eukaryotic systems, suggesting that all lipoyl synthases recognize their H-protein counterparts similarly.^59^ Furthermore, a comparison of the LIAS-H-protein complex with computationally predicted complexes of LIAS and the human E2 subunits suggested that productive interactions between LIAS and E2 subunits are unlikely, reinforcing the central role of GCSH as the sole lipoylation substrate in humans. Consistent with this architecture, humans lack a dedicated LC salvage pathway, and exogenous LA fails to restore lipoylation or ameliorate clinical phenotypes associated with LC deficiency in patients.^24,49–51^

After the generation of the mature cofactor, the amidotransferase LIPT1 distributes the lipoyl group from GCSH to the other LCPs.^55^ However, early pathway models incorrectly assigned LIPT1 (and LIPT2) as a lipoate ligase due to its shared structural scaffold with bacterial LplA, thus leading to incorrect mechanistic proposals in which free lipoic acid could be directly incorporated into mitochondrial proteins. Recognition that LIPT1 instead catalyzes lipoyl-group transfer between protein substrates established GCSH as the central lipoyl donor in human metabolism and fundamentally revised the understanding of mitochondrial lipoylation. Consequently, GCSH serves as the hub of human LA biosynthesis, functioning as both the sole substrate for LIAS and the donor from which the mature cofactor is ultimately distributed by LIPT1 to all other lipoyl-dependent proteins.

### Octanoyltransferase Catalyzes the First Committed Step of LA Biosynthesis

In the first committed step of LA biosynthesis, the octanoyltransferase, LipB (LIPT2 in *H. sapiens*, Lip2 in *B. subtilis*, *S. aureus*, and *S. cerevisiae*), catalyzes the transfer of an octanoyl group from the octanoyl acyl-carrier protein (octanoyl-ACP) to the conserved lysine residue of the LCP, thereby coupling fatty acid biosynthesis to *de novo* lipoic acid assembly. The physiological role of LipB was established through *in vitro* labeling studies with [^35^S] lipoic acid and [^14^C] octanoic acid, where a strain of *E. coli* lacking LplA retained substantial lipoylation of 2-oxoacid dehydrogenase complexes, demonstrating the existence of a biosynthetic pathway independent of the salvage pathway.^60–62^ Indeed, it was later identified that LipB was the enzyme responsible for initiating the first committed step of *de novo* lipoic acid biosynthesis. *In vitro* reconstitution of LipB demonstrated that the octanoyl group from ACP was directly transferred to the LCP.^63^ Subsequent mechanistic studies of LipB revealed that, upon treatment with 5,5’-dithiobis­(2-nitrobenzoic acid) as a function of time, one of its three highly conserved cysteinyl residues reacted at a rate 50-fold greater than the others, suggesting a role in covalent catalysis.^64^ Site-directed mutagenesis and kinetic analyses further established that this strictly conserved cysteine forms a transient acyl-enzyme thioester intermediate.^65^ Structural characterization of *M. tuberculosis* LipB (Figure 3A) identified a conserved lysine positioned adjacent to the catalytic cysteine, supporting a cysteine/lysine dyad mechanism for acyl transfer.^66^

**Figure 3:**
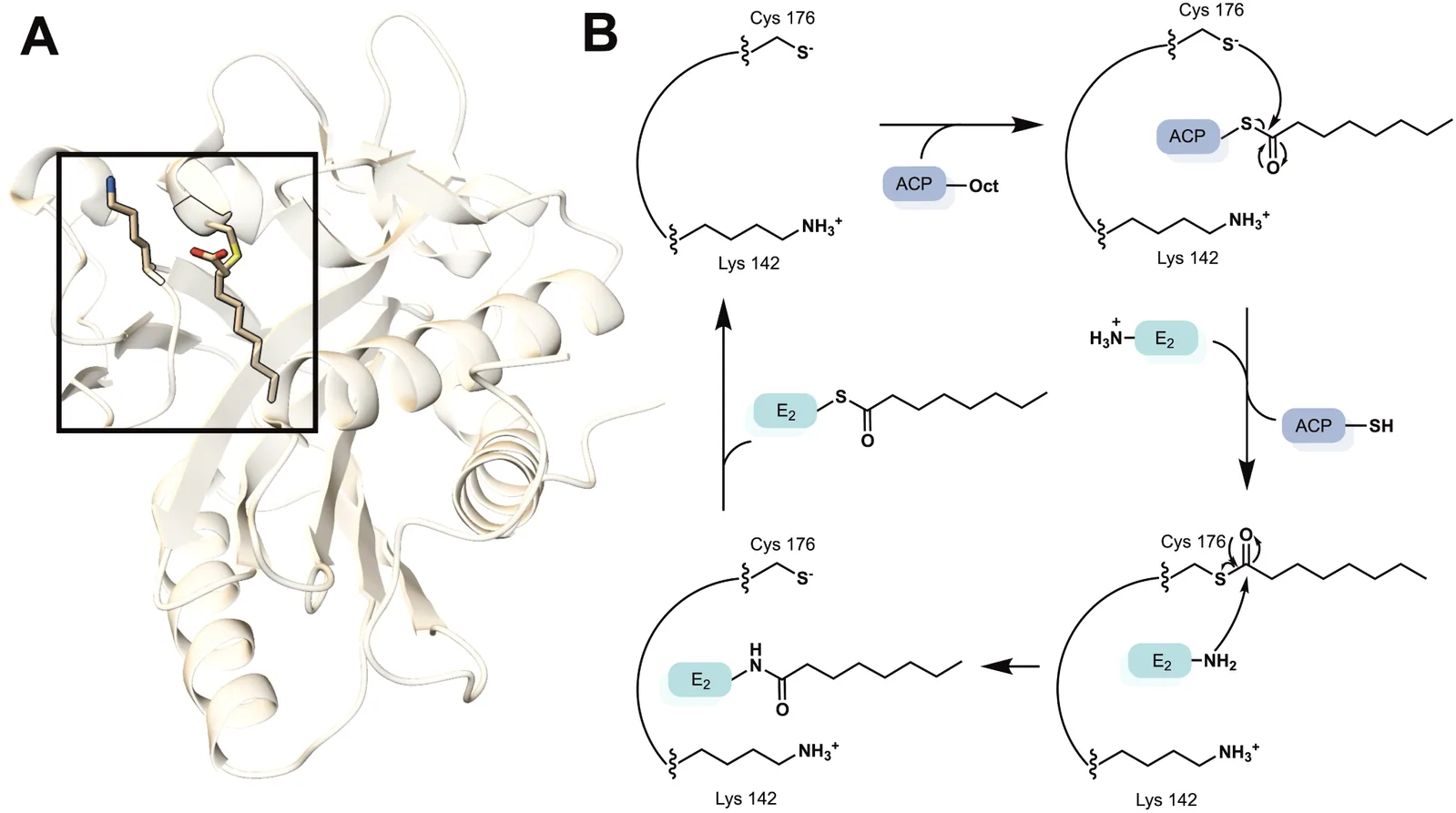
Octanoyltransferase uses a cysteine/lysine dyad mechanism for acyl transfer. (A) X-ray crystal structure of the octanoylated intermediate of LipB from *M. tuberculosis* at 1.08 Å (PDB: 1W66). The cysteine (C176) and lysine (K142) active site facial dyad is boxed in black, with the cysteine residue modified with an octanoyl group that is off-pathway of normal LipB synthesis (i.e., adduct formation). The structure was visualized and colored in ChimeraX. (B) Proposed ping-pong mechanism of the octanoylation by LipB. In the first step, LipB utilizes C176 to perform a nucleophilic attack of the octanoyl group from octanoyl-ACP to generate a covalent thioester intermediate and release holo-ACP. The appropriate receiver LCP then binds; in this example, the E_2_ subunit of PDH. The ε-amino group lysine side chain of the receiver LCP attacks the thioester bond of the LipB-octanoyl intermediate, yielding the octanoyl-LCP. At this step, it is hypothesized that proton shuttling between K142 from LipB and the receiver LCP (not shown) is required to generate the nucleophilic species. Finally, the octanoylated receiver LCP can dissociate, freeing the LipB cysteine residue for subsequent turnover.

Together, the available biochemical, structural, and mutagenesis evidence suggests a ping-pong mechanism for octanoyl transfer (Figure 3B). LipB first accepts the octanoyl group from octanoyl-ACP, forming a covalent thioester intermediate and releasing holo-ACP. The LCP then binds, and its target lysine side chain attacks the thioester bond of the LipB-octanoyl intermediate, yielding octanoyl-LCP and freeing the LipB cysteine residue for subsequent turnover. It was postulated that proton transfers to and from substrates and products are also mediated by this conserved lysine. Site-directed mutagenesis also verified that the conserved cysteine and lysine residues are essential for catalysis. Finally, the product of this reaction, octanoyl-LCP, can then be converted to lipoyl-LCP by LipA.

### Lipoyl Synthase: A Member of Radical *S*-Adenosylmethionine Superfamily

In the next step of LA biosynthesis, lipoyl synthase catalyzes two kinetically challenging steps: the installation of two sulfur atoms at C6 and C8 of the octanoyl group.^2,36,67,68^ This transformation requires cleavage of two unactivated C–H bonds and stereoselective formation of C–S bonds at aliphatic carbon centers.^69^ Initial metabolic labeling studies established that only the hydrogen atoms replaced by sulfur are removed during the reaction and further showed that sulfur insertion at C6 proceeds with inversion of configuration.^70,71^ Early metabolic feeding experiments also demonstrated that hydroxylated octanoate analogues, such as 6-hydroxyoctanoic acid, 8-hydroxyoctanoic acid, or 6,8-dihydroxyoctanoic acid, were not incorporated into LA, effectively excluding pathways involving the initial oxygenation of the substrate. By contrast, 6-thiooctanoic acid or 8-thiooctanoic acid was converted to LA in *E. coli*, consistent with direct sulfur insertion into the octanoyl precursor.^72^ Although these studies initially suggested that sulfur insertion at C8 precedes insertion at C6, subsequent mechanistic work established that the order of sulfur incorporation is reversed.

LipA belongs to the radical *S*-adenoylmethionine (SAM) superfamily, a broad class of enzymes that catalyze chemically challenging transformations through the intermediacy of 5′-deoxyadenosyl 5’-radicals (5′-dA•).^73,74^ Almost all radical SAM (RS) enzymes contain a CX_3_CX_2_C motif, in which the three indicated cysteines bind three iron ions of an essential [4Fe-4S] cluster (also known as [4Fe-4S]_RS_).^75^ This cysteine-containing motif is located within the RS domain of LipA, which adopts a partial (βα)_6_ triosephosphate isomerase (TIM)-barrel fold.^76^ The unique fourth iron (Fe_α_) of the cluster is bound by SAM in a bidentate fashion (Figure 4A). Upon one-electron reduction of the cluster by physiological reducing systems, such as the flavodoxin/flavodoxin reductase system in *E. coli*,^77,78^ or chemical reductants, including dithionite,^39,79^ the cluster induces the reductive cleavage of SAM to generate methionine and the highly reactive 5′-dA• (Figure 4B). This radical abstracts a hydrogen atom (H•) from the octanoyl substrate to initiate sulfur attachment.

**Figure 4:**
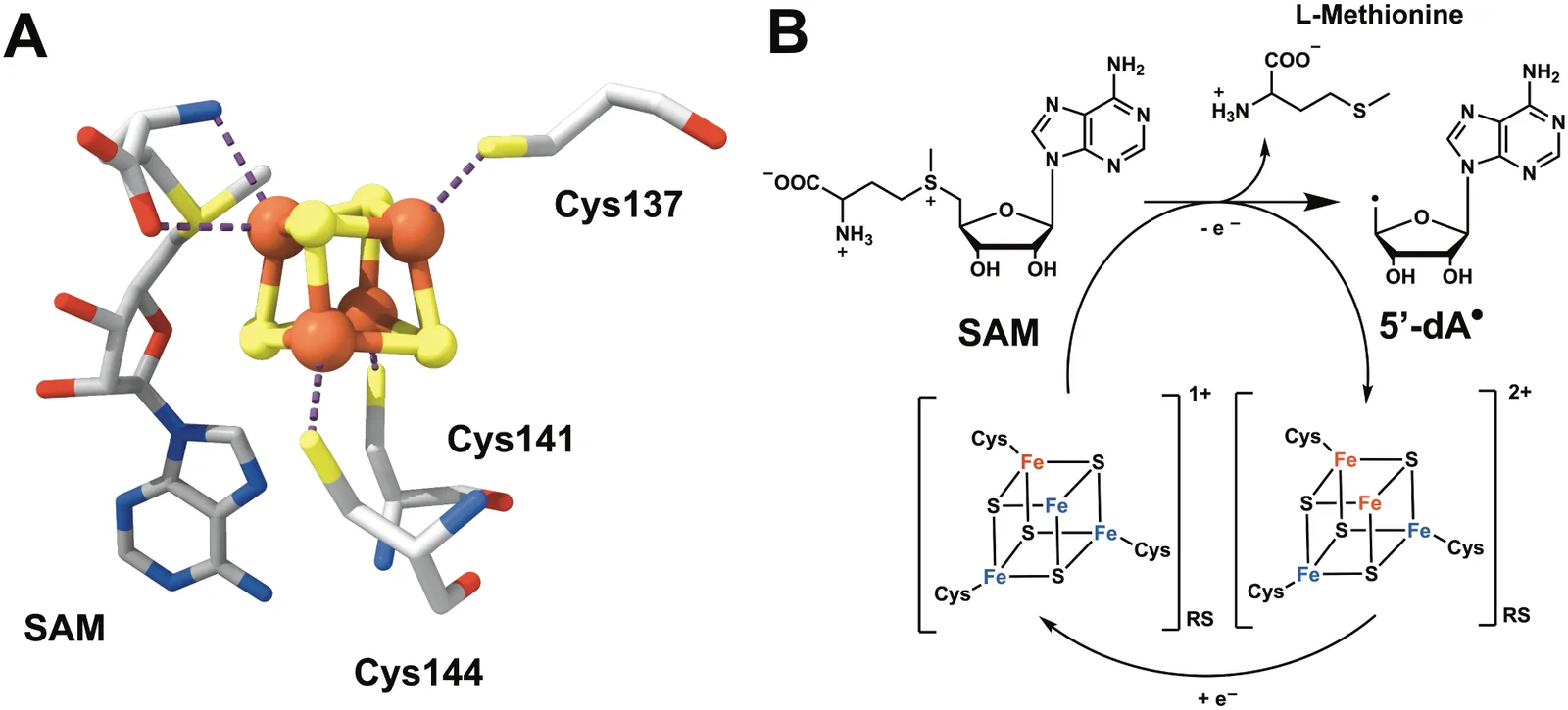
(A) Coordination of the radical SAM [4Fe–4S] cluster by SAM. In all canonical lipoyl synthases, three of the RS cluster iron ions are ligated by cysteinyl residues found in the highly conserved CX_3_CX_2_C motif. The remaining “unique” iron is coordinated by a molecule of SAM in a bidentate fashion, facilitating its reductive cleavage. Pictured above is the [4Fe–4S]_RS_ cluster from human LIAS (PDB 8 V0J). Models were visualized and colored in ChimeraX. (B) General mechanism for the homolytic cleavage of SAM. RS enzymes coordinate a characteristic [4Fe–4S]_RS_ cluster, which, in its reduced state, can inject an electron into the sulfonium of the SAM molecule, inducing its homolytic cleavage to l-methionine and 5′-dA•. For the RS cluster pictured here, the Fe^2+^ ions are colored in blue, and the Fe^3+^ ions are colored in red.

LipA belongs to a subclass of radical SAM enzymes that contain one or more auxiliary iron–sulfur clusters in addition to the canonical radical SAM cluster.^27^ In LipA^30^ and the closely related biotin synthase,^80,81^ the auxiliary clusters serve as the immediate sulfur donor. During catalysis, sulfur atoms are transferred from this cluster to the substrate, degrading the auxiliary cluster ([4Fe-4S]_aux_) and inactivating the enzyme after a single turnover. Restoration of catalytic activity, therefore, requires cluster reassembly and repair. Ultimately, the use of a sacrificial Fe/S cluster provides a direct solution to the longstanding question of how sulfur is activated and appended during LA biosynthesis.

### Mechanistic and Structural Elucidation of Canonical Lipoyl Synthases

Elucidation of the lipoyl synthase mechanism depended on the development of active preparations of LipA. Early attempts to isolate *E. coli* LipA yielded protein containing approximately 2 mol of iron and sulfide per mol of polypeptide, with little or no detectable activity.^82,83^ Robust catalytic activity was achieved only after coexpression of LipA with the iron–sulfur cluster (*isc*) maturation genes from *Azotobacter vinelandii* and purification under strictly anaerobic conditions, establishing the importance of complete cluster loading for catalysis.^28,35,67,84^ Bioinformatic and spectroscopic characterization of the active enzyme demonstrated that *E. coli* LipA contained two distinct [4Fe-4S] clusters.^36^ LipA contains six strictly conserved cysteine residues; in addition to the CX_3_CX_2_C motif characteristic of almost all RS enzymes, a second CX_4_CX_5_C motif is usually present in the N-terminal region of the polypeptide and is conserved amongst all canonical lipoyl synthases. When wild-type *E. coli* LipA and variants containing substitutions at all of the cysteine ligands were expressed and reconstituted with ^57^Fe after purification, Mössbauer spectroscopy showed the presence of two [4Fe–4S] clusters: the [4Fe-4S]_RS_ cluster and the [4Fe-4S]_aux_ cluster.^36^ Subsequent biochemical studies indicated that 2 equiv of SAM were required to form 1 equiv of LA, consistent with sequential hydrogen-atom abstraction at C6 and C8 of the octanoyl substrate.^67^

A central mechanistic question concerned the source of the two sulfur atoms incorporated in the LC (Figure 5). Isotopic labeling experiments using ^34^S-enriched and natural abundance (^32^S) LipA demonstrated that both sulfur atoms originate from the same polypeptide in the absence of an exogenous sulfur donor, suggesting that the [4Fe-4S]_aux_ cluster serves as the immediate sulfur source.^68^ Electron paramagnetic resonance (EPR) and hyperfine sublevel correlation (HYSCORE) spectroscopies with ^57^Fe-labeled enzyme captured a substrate radical—originating from a stabilized conjugated 2,4-hexadienoyl-containing substrate analog—positioned in direct contact with an Fe/S cluster (presumably the [4Fe-4S]_aux_), providing strong evidence for an Fe/S cluster as the sulfur source.^23,85,86^ Subsequent isolation of a catalytically competent monothiolated intermediate and analysis by Mössbauer spectroscopy revealed a covalent linkage between C6 of the substrate and a partially degraded [3Fe-4S] cluster,^87^ later shown to be the auxiliary cluster.^76^ This intermediate accumulates when the substrate is deuterated at C8, indicating an elevated kinetic isotope effect associated with H• abstraction at C8.^67,69^ Moreover, under normal turnover, sulfur attachment at C6 is significantly faster than attachment at C8, allowing for the intermediate to accumulate when the enzyme is incubated under turnover conditions with only 1 equiv of SAM. This characteristic enabled the intermediate to be isolated and reintroduced into a reaction, where it proceeded to product at a rate approximately equal to that of the overall turnover.

**Figure 5:**
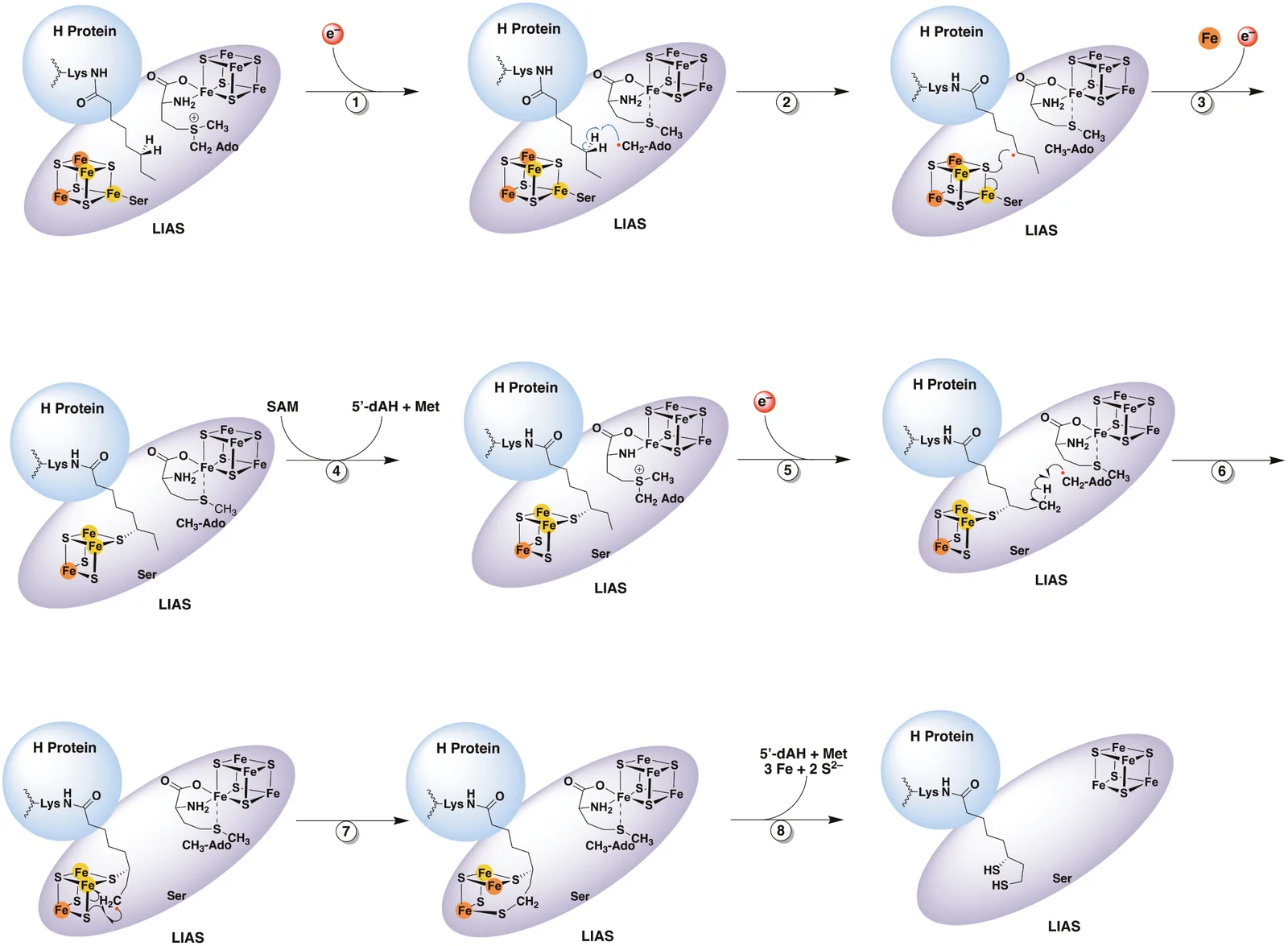
Proposed mechanism of lipoyl synthase catalysis. Reductive cleavage of SAM at the [4Fe-4S]_RS_ cluster (1) generates a 5′-dA•, which abstracts the C6 pro-*R* hydrogen atom from the octanoyllysyl substrate (2). The resulting substrate radical attacks a μ_3_-sulfido ligand of the [4Fe-4S]_aux_ cluster (3), leading to sulfur insertion and cluster reduction. Subsequent loss of Fe^2+^ and electron transfer yields a C6 sulfur-inserted intermediate coordinated to a [3Fe-4S]-like cluster. A second equivalent of SAM (4) is then cleaved by the RS cluster (5) to generate a second equivalent of 5′-dA•, which abstracts a hydrogen atom from C8 of the intermediate (6). Radical attack on a μ_2_-sulfido ligand of the auxiliary cluster results in insertion of the second sulfur atom (7). Protonation and product release afford the lipoyl cofactor in its reduced (dihydrolipoyl) form (8). Once the mature LC is made, it can be installed on protein complexes responsible for respiration and metabolism.

Structural studies have also provided atomic-level views of the catalytic cycle. The first crystal structure of LipA from *Thermosynechococcus elongatus* at 1.6 Å resolution revealed an N-terminal domain that binds the [4Fe-4S]_aux_ cluster, a central RS domain, and an elongated C-terminal segment containing an RSSY motif, which is highly conserved among canonical lipoyl synthases (Figure 6A).^88^ The second serine residue within this motif coordinates the unique iron of the auxiliary cluster, an unusual ligand for an Fe/S cluster. A subsequent crystal structure of LipA from *Mycobacterium tuberculosis* provided further evidence for the intermediate, as observed by Mössbauer spectroscopy, showing a [3Fe-4S] cluster covalently attached to C6 of the substrate.^76,79^ Moreover, the structure showed methionine still coordinated to the [4Fe-4S]_RS_ cluster, with C5’ of 5’-dAH 4.2 Å from the sulfur of methionine and 3.9 Å from C6 of the 6-mercaptooctanoyl species, perfectly agreeing with the hypothesized mechanism of catalysis.^76^ In addition, the structure provided a rationale for the degradation of the [4Fe-4S]_aux_ cluster to a [3Fe-4S]_aux_ cluster, as loss of the iron ion would be necessary for the substrate radical at C8 to attack one of the remaining sulfide ions of the cluster in the subsequent step of catalysis^76^ (Figure 6A, *inset*). Most recently, a structure of human LIAS (Figure 6B) captured multiple catalytic states, including complexed with the glycine cleavage H protein substrate directly ligated to the partially degraded [3Fe-4S]_aux_ cluster.^59^ These studies revealed substantial conformational rearrangements that shorten the distance between the RS and auxiliary clusters (from 13.7 Å to 11.8 Å) and close the substrate channel, thereby promoting efficient radical generation and sulfur transfer (Figure 6B, *inset*).

**Figure 6:**
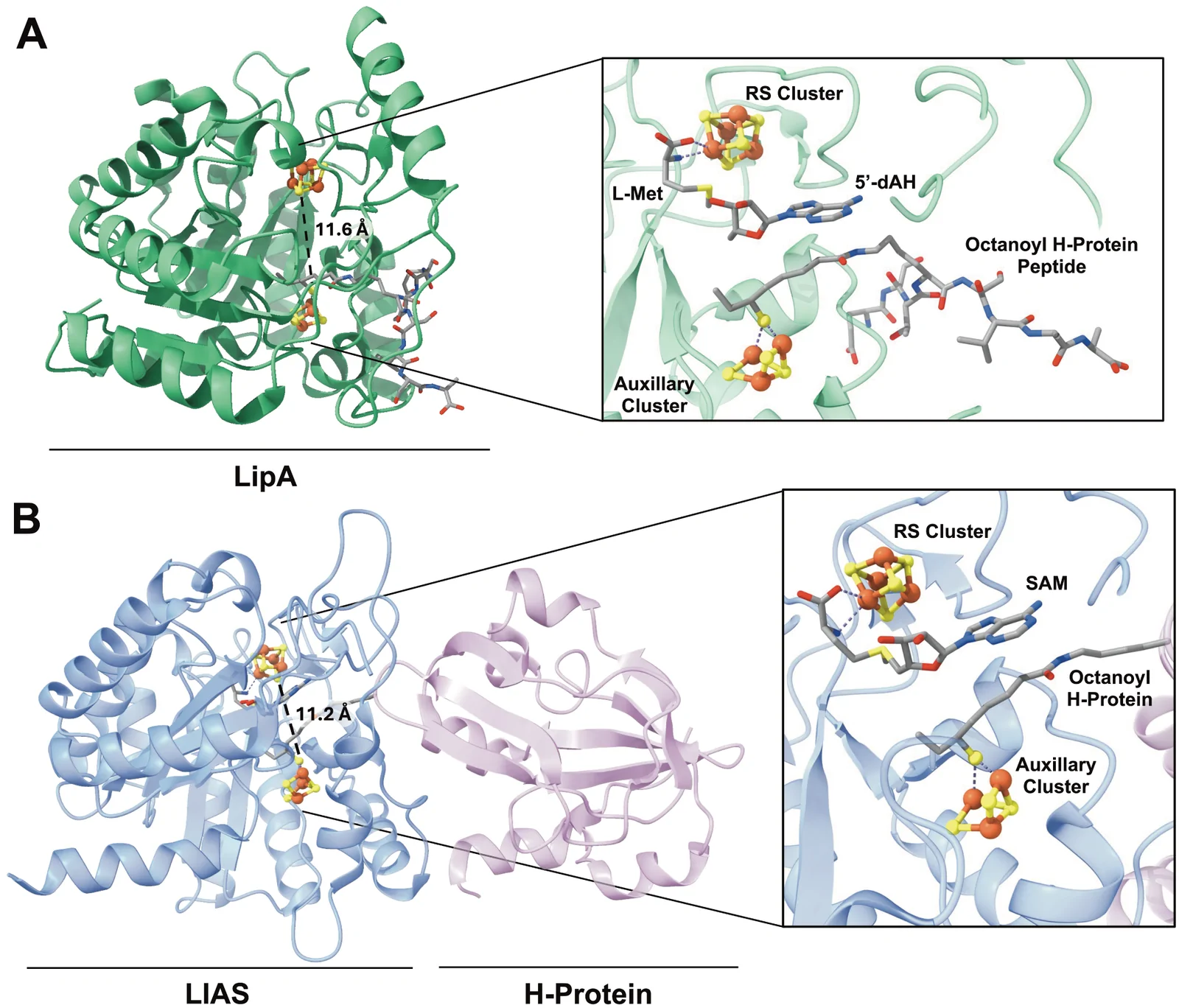
Crystal structure of crosslinked intermediate in lipoyl synthase. Crystal structures of (A) *Mycobacterium tuberculosis* LipA (PDB 5EXK) at 1.86 Å and (C) human LIAS (8UGO) at 2.45 Å, each reacted with 1 equiv of SAM and either an octanoyl-peptide (A, *inset*) or octanoyl-H-protein (B, *inset*) substrate. Each structure reveals a [3Fe-3S-1­(6S)-thio-octanoamide] LCP intermediate, confirming that sulfur insertion first happens on C6 of the octanoyl substrate. Structures were visualized and colored in ChimeraX.

Collectively, these studies establish that lipoyl synthases contain two [4Fe-4S] clusters with distinct functions: the RS cluster generates radical intermediates, whereas the auxiliary cluster donates both sulfur atoms required for forming the LC. Sulfur transfer proceeds through a cluster-bound monothiolated intermediate and is accompanied by the degradation of the auxiliary cluster, which inactivates the enzyme. Further turnover requires that the auxiliary cluster be reassembled or repaired during turnover. How the unique iron ion is lost during catalysis and how the auxiliary cluster is restored during or after turnover are outstanding questions that are under investigation.

### Noncanonical Dual-Enzyme Lipoyl Synthases

The long-standing view that LC biosynthesis proceeds exclusively through a single canonical lipoyl synthase has recently been challenged by the discovery of an alternative pathway in *T. kodakarensis*.^39,40^ In this system, sulfur attachment is catalyzed sequentially by two radical SAM enzymes, LipS1 and LipS2 (Figure 7), previously annotated as biotin synthases. Similarly to biotin synthase,^81^ LipS2 first attaches sulfur at C8 of the octanoyl substrate, while LipS1 subsequently attaches sulfur at C6. LipS2 and LipS1 each contain both an RS and an auxiliary cluster but differ from canonical LipAs in the amino acid motif for binding the auxiliary cluster. In particular, the conserved serine ligand of the canonical RSSY motif is replaced with a histidine in LipS1, which is proposed to fulfill an analogous role in cluster coordination and sulfur transfer. Despite this altered active-site architecture, both enzymes appear to employ a sacrificial auxiliary cluster to donate sulfur atoms, suggesting that cluster consumption is a general solution to sulfur attachment during LA biosynthesis. Bioinformatic analysis^39,40^ suggests that this mechanism is found in most archaea and some bacteria, demonstrating that nature has evolved multiple strategies to accomplish sulfur attachment to unactivated carbon centers while preserving the core mechanistic principles of radical initiation and auxiliary-cluster-mediated sulfur donation.

**Figure 7:**
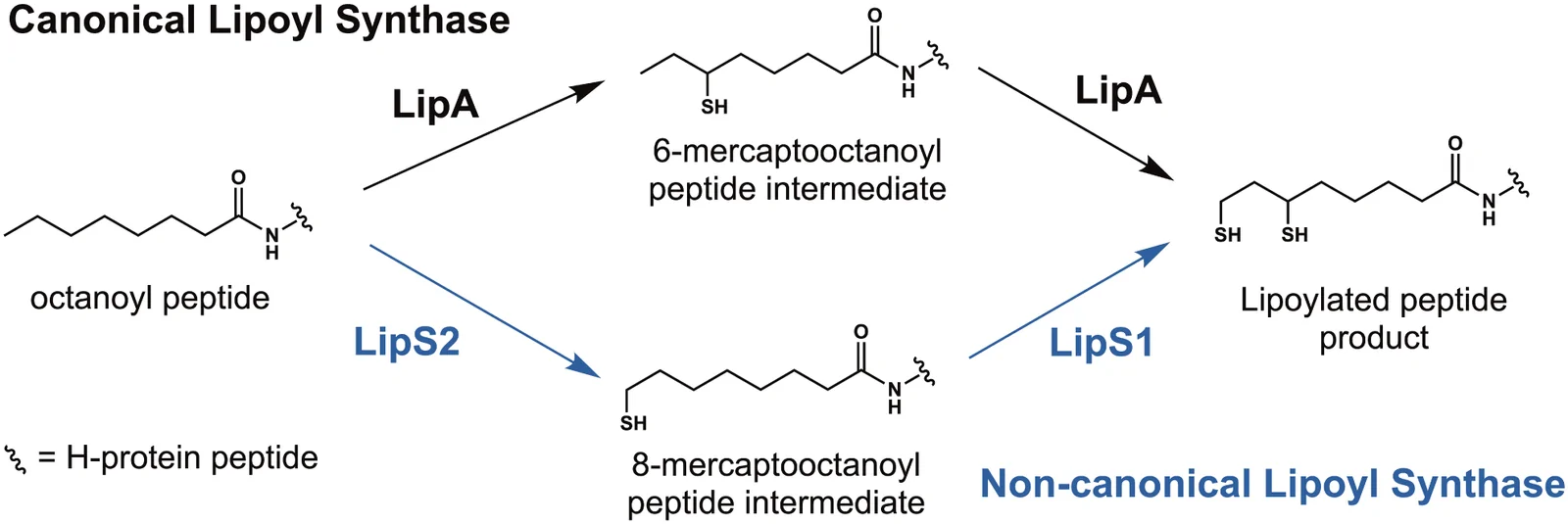
Noncanonical lipoyl synthase systems from *T. kodakarensis*. In canonical lipoic acid synthesis (*top*), lipoyl synthases like *E. coli* LipA catalyze the stepwise addition of sulfur at C6 followed by C8, using sulfur donated from the auxiliary cluster. In contrast, noncanonical lipoic acid synthesis (*bottom*) requires two enzymes: LipS2 and LipS1. LipS2 acts only on an octanoyl-containing substrate, while LipS1 acts only on an 8-mercaptooctanoyl-containing substrate. Therefore, similar to the *E. coli* biotin synthase BioB and in contrast to canonical lipoyl synthases, sulfur attachment takes place initially at the terminal C8 and then at the C6 methylene carbon.

### Regeneration of Lipoyl Synthase for Catalysis: The Missing Piece

A defining feature of lipoyl synthase is that its auxiliary cluster is consumed during sulfur attachment, rendering the enzyme inactive after a single turnover unless the cluster is reformed. This mechanistic requirement accounts for the long-standing observation that early *in vitro* assays^28,30,56^ produced no more than approximately one equivalent of lipoylated product per enzyme molecule. The resulting question was whether lipoyl synthase functions as a true catalyst *in vivo* or whether the protein is degraded and resynthesized after each turnover. Ribosome profiling analyses and estimates of demands for cellular lipoylation strongly support the conclusion that lipoyl synthase must be catalytic *in vivo*,^89^ necessitating a mechanism for the regeneration of the sacrificial auxiliary cluster.

The existence of dedicated iron–sulfur cluster assembly and trafficking pathways in all domains of life suggested that cluster carrier proteins might restore catalytic competence to lipoyl synthase.^90–92^ In humans, independent clinical studies identified pathogenic variants in the Fe/S cluster chaperones NFU1 and BOLA3 that are associated with severe metabolic diseases characterized, in part, by impaired LA synthesis.^93,94^ These observations implicated mitochondrial Fe/S trafficking factors in maintaining human LIAS activity. Biochemical studies have established that the *E. coli* NFU1 homolog, NfuA, directly repairs the auxiliary cluster of LipA.^30^ Cluster-loaded NfuA forms a stable complex with LipA and transfers a [4Fe-4S] cluster to the enzyme in a process that is not rate-limiting during catalysis. Isotopic labeling with ^34^S-enriched NfuA has demonstrated that sulfur atoms from the transferred cluster are subsequently incorporated into the LC, providing direct evidence that NfuA restores the sacrificial sulfur donor required for continued catalytic turnover. Moreover, a modified peptide with natural abundance ^32^S was produced for 2 equiv before ^34^S-enriched peptide was detected, suggesting that the auxiliary cluster is fully consumed before NfuA regenerates the cluster (Figure 8).^30^ However, a Δ*nfuA* mutant in *E. coli* shows no prominent growth defect or lipoylation deficiency, suggesting the presence of other cluster-regeneration factors *in vivo*. In the absence of NfuA, the scaffold protein IscU was also observed *in vitro* to transfer a cluster to LipA, indicating that multiple components of the iron–sulfur assembly machinery are capable of reconstituting the auxiliary cluster.^30^

**Figure 8:**
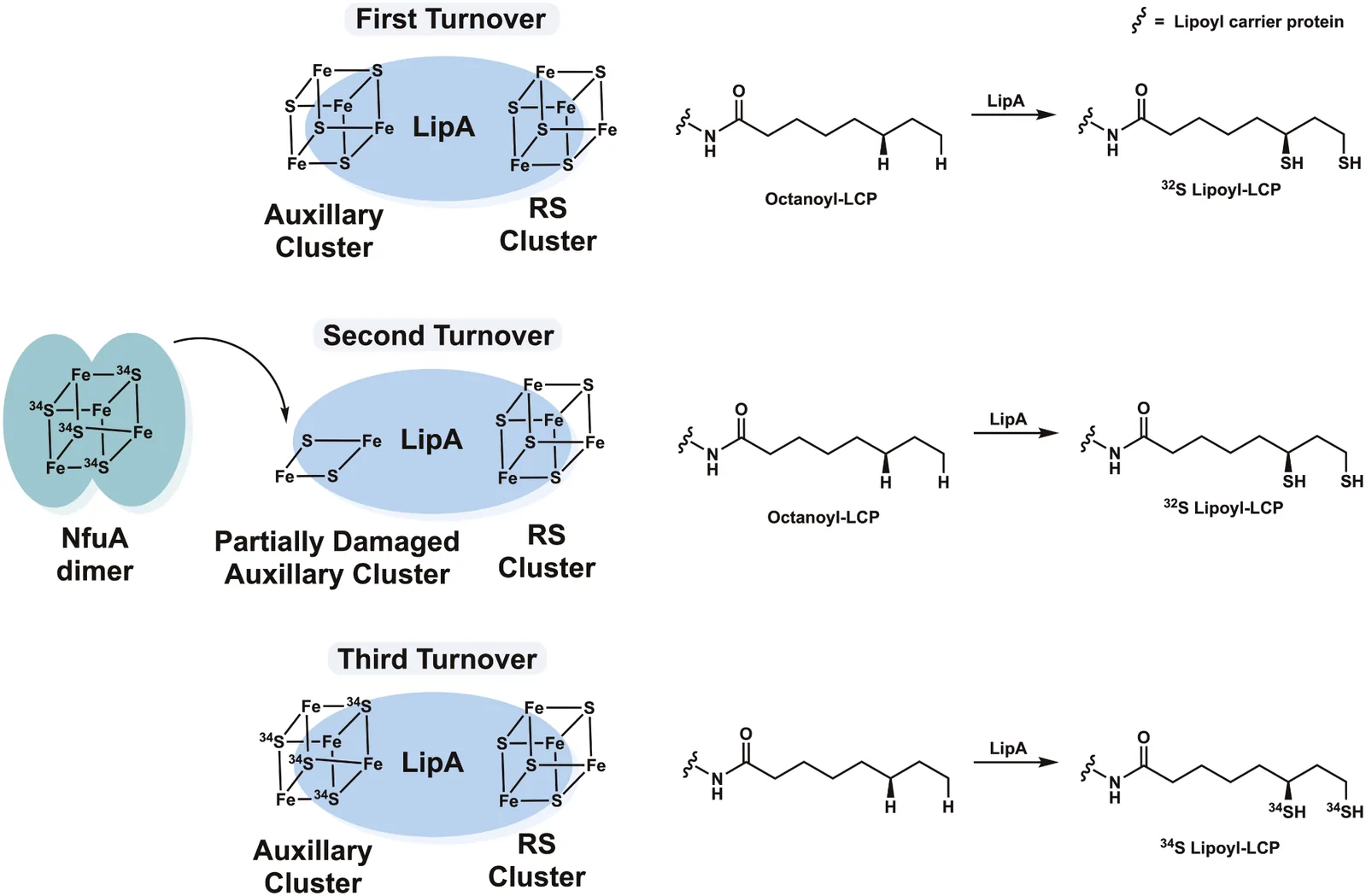
Proposed *E. coli*LipA [4Fe-4S]_aux_ cluster regeneration by *E. coli* *NfuA*. During activity assays utilizing natural abundance (^32^S) [4Fe-4S] LipA and ^34^S-labeled [4Fe-4S] NfuA, two equivalents of unlabeled (^32^S) lipoyl product were observed before the subsequent formation of ^34^S-labeled lipoyl product, suggesting that two turnovers are required before incorporation of the labeled cluster from NfuA. As such, in the presence of NfuA, LipA is able to utilize all four sulfide ions from the cluster into the lipoyl product before cluster repair.

An analogous mechanism operates in the human mitochondrial system. Reconstituted human NFU1 binds a [4Fe-4S] cluster, forms a tight complex with LIAS, and efficiently restores enzyme activity, indicating that auxiliary cluster repair is conserved from bacteria to humans.^56^ This finding provides a molecular explanation for the profound LA deficiency observed in patients harboring the NFU1 variants. In contrast, the inclusion of BOLA3 does not significantly stimulate LIAS turnover *in vitro*, and its precise role remains unresolved.^95^ Additional studies^96^ indicate that ISCA1 can cooperate with NFU1 and may participate in the delivery of clusters to LIAS or in the maintenance of the RS cluster. Collectively, these findings establish that lipoyl synthase is a bona fide catalyst whose activity depends on the continuous repair of its auxiliary [4Fe-4S] cluster by the cellular iron–sulfur trafficking machinery. Cluster transfer from proteins such as IscU and NfuA/NFU1 resolves the long-standing paradox posed by sacrificial sulfur donation and completes the mechanistic framework for *de novo* LA biosynthesis.

### Regulation of LC Biosynthesis by Ferredoxin-Dependent Electron Transfer

Early biochemical studies established that reduced ferredoxins and flavodoxins can support turnover *in vitro*, consistent with the general requirement of RS enzymes for one-electron reductants. However, the extent to which specific electron carriers regulate LipA activity *in vivo* remained unclear. Recent studies^57,97^ have established ferredoxin 1 (FDX1) as a central regulator of mitochondrial lipoic acid biosynthesis, extending its functional role beyond its canonical involvement in steroid biosynthesis.^98^ Loss of FDX1, but not its paralog ferredoxin 2 (FDX2), abolishes lipoylation of all the 2-oxoacid dehydrogenase complexes—a similar phenotype observed with LIAS loss-of-function mutations.^23,99,100^ Cellular dependency analyses further revealed a strong genetic association between FDX1 and LIAS, supporting a functional partnership within the lipoic acid biosynthetic machinery.

*In vitro* mechanistic studies have shown that FDX1 directly interacts with LIAS and functions as its physiological electron donor during sulfur insertion.^97^ Biochemical and cellular interaction assays demonstrated that FDX1 promotes the productive association between LIAS and its lipoyl carrier substrates, enabling reductive activation of SAM. In the absence of FDX1, the formation of catalytically competent LIAS is abolished, resulting in a complete loss of lipoylation despite stabilization and accumulation of the LIAS protein itself. Together, these findings support a model in which FDX1 serves as a dedicated electron-transfer partner that channels reducing equivalents to LIAS, thereby coupling mitochondrial redox metabolism to lipoic acid biosynthesis. Intriguingly, recent work^101^ demonstrated that although hypoxia fails to restore lipoylation in either FDX1- or LIAS-deficient cells, it rescues cellular growth defects, revealing that lipoate-dependent metabolism can become dispensable under low oxygen conditions.

### Perspective

Over the past few decades, substantial progress has been made in elucidating the biosynthesis of LAs. Since its initial isolation from large quantities of liver tissue in the 1950s,^17^ advances in recombinant protein production, mass spectrometry, and other biochemical techniques have transformed both our understanding of LA biosynthesis and our appreciation of its therapeutic potential. A major advance came when multiple turnovers of the *E. coli* LipA were observed *in vitro* for the first time. Although this enzyme sacrifices its [4Fe-4S]_aux_ cluster as the sulfur source during catalysis, this cluster can be reconstituted efficiently by the iron–sulfur cluster carrier NfuA in a step that is not rate-limiting to catalysis. In a subsequent study, the analogous human orthologue NFU1 efficiently reconstituted the LIAS auxiliary cluster and supported multiple turnovers *in vitro*, suggesting a conserved role across multiple kingdoms of life. However, the molecular basis by which lipoyl synthase is recognized and targeted for cluster regeneration *in vivo* remains poorly understood.

Despite these advances, several fundamental questions remain unanswered. One question concerns the fate of the unique iron released upon formation of the monothiolated intermediate as well as that of the remaining iron at the conclusion of catalysis (Figure 5). Structural studies of lipoyl synthase have revealed a solvent-accessible channel connecting the protein surface to the auxiliary cluster binding site, suggesting a potential pathway for iron release during turnover and preventing the accumulation of labile iron near the radical-generating center. It remains unclear whether the released iron is recaptured during cluster regeneration or instead enters the cellular labile-iron pool. In addition, the state of the cluster during regeneration also remains undefined: whether a complete [4Fe-4S] cluster is transferred in a single event or, alternately, if some processive repair mechanism exists. Important uncertainties also extend to newly identified noncanonical lipoyl synthases, LipS1 and LipS2, for which mechanisms of cluster regeneration have not yet been demonstrated both *in vitro* and *in vivo*.

Finally, although pathogenic variants in BOLA3 cause LA deficiency phenotypes, implying this protein in LA biosynthesis, its precise molecular function remains unknown. Current evidence suggests that BOLA3 participates in mitochondrial Fe/S-cluster trafficking and homeostasis. However, whether it acts directly in auxiliary cluster delivery to LIAS or functions indirectly through upstream Fe/S transfer pathways has yet to be established. Resolving this question will be essential for defining the molecular basis of Fe/S cluster repair during lipoyl cofactor biosynthesis. Taken together, these studies have established a mechanistic framework for understanding LA biosynthesis while highlighting key unresolved questions in iron–sulfur cluster trafficking, enzyme repair, and human disease. Addressing these issues will not only deepen our understanding of RS enzymology but also provide new insights into the molecular basis of disorders associated with impaired LA metabolism.

## Abbreviations

SAM: *S*-adenosyl-methionine
RS: radical SAM
*E. coli*: *Escherichia coli*
LipA: lipoyl synthase (bacterial)
LIAS: lipoyl synthase (human)
LCP: lipoyl carrier protein
LC: lipoyl cofactor
PDC: pyruvate dehydrogenase complex
KDC: α-ketoglutarate dehydrogenase complex
BCOADC: branched-chain oxo-acid dehydrogenase complex
GCS: glycine cleavage system
ADC: acetoin dehydrogenase complex
Fe/S: iron–sulfur
LplA: lipoate protein ligase A
ACP: acyl carrier protein
GCSH: glycine cleavage system H-protein
5′-dA: 5’-deoxyadenosyl radical
TIM: triosephosphate isomerase
*Isc*: iron–sulfur cluster maturation system
EPR: electron paramagnetic resonance
HYSCORE: hyperfine sublevel correlation spectroscopy.
